# Relationship Between Physical Activity and Chronic Musculoskeletal Pain Among Community-Dwelling Japanese Adults

**DOI:** 10.2188/jea.JE20140025

**Published:** 2014-11-05

**Authors:** Masamitsu Kamada, Jun Kitayuguchi, I-Min Lee, Tsuyoshi Hamano, Fumiaki Imamura, Shigeru Inoue, Motohiko Miyachi, Kuninori Shiwaku

**Affiliations:** 1 Department of Health Promotion and Exercise, National Institute of Health and Nutrition, Shinjuku-ku, Tokyo, Japan; 1 独立行政法人国立健康・栄養研究所健康増進研究部; 2 Japan Society for the Promotion of Science, Chiyoda-ku, Tokyo, Japan; 2 日本学術振興会; 3 Physical Education and Medicine Research Center UNNAN, Unnan, Shimane, Japan; 3 身体教育医学研究所うんなん; 4 Division of Preventive Medicine, Brigham and Women’s Hospital, Harvard Medical School, Boston, MA, USA; 4 Division of Preventive Medicine, Brigham and Women’s Hospital, Harvard Medical School; 5 Center for Community-based Health Research and Education (COHRE), Organization for the Promotion of Project Research, Shimane University, Izumo, Japan; 5 島根大学研究機構戦略的研究推進センター; 6 MRC Epidemiology Unit, Institute of Metabolic Science, University of Cambridge School of Clinical Medicine, Cambridge, UK; 6 MRC Epidemiology Unit, Institute of Metabolic Science, University of Cambridge School of Clinical Medicine; 7 Department of Preventive Medicine and Public Health, Tokyo Medical University, Shinjuku-ku, Tokyo, Japan; 7 東京医科大学公衆衛生学講座; 8 Department of Environmental and Preventive Medicine, Shimane University School of Medicine, Izumo, Shimane, Japan; 8 島根大学医学部環境予防医学

**Keywords:** exercise, musculoskeletal pain, arthritis, epidemiology, public health

## Abstract

**Background:**

Both little and excessive physical activity (PA) may relate to chronic musculoskeletal pain. The primary objective of this study was to characterize the relationship of PA levels with chronic low back pain (CLBP) and chronic knee pain (CKP).

**Methods:**

We evaluated 4559 adults aged 40–79 years in a community-based cross-sectional survey conducted in 2009 in Shimane, Japan. We used self-administered questionnaires to assess sociodemographics and health status: PA was assessed by the International Physical Activity Questionnaire, and CLBP and CKP were assessed by a modified version of the Knee Pain Screening Tool. We examined relationships of PA with prevalence of CLBP and CKP using Poisson regression, controlling for potential confounders.

**Results:**

CLBP and CKP were both prevalent (14.1% and 10.7%, respectively) and associated with history of injury, medication use, and consultation with physicians. PA was not significantly related to CLBP or CKP (*P* > 0.05) before or after adjustment for potential confounders. For example, compared with adults reporting moderate PA (8.25–23.0 MET-hours/week), prevalence ratios for CKP adjusted for sex, age, education years, self-rated health, depressive symptom, smoking, chronic disease history, and body-mass index were 1.12 (95% confidential interval [CI] 0.84–1.50) among those with the lowest PA and 1.26 (95% CI 0.93–1.70) among those with the highest PA (*P* quadratic = 0.08). The prevalence ratios were further attenuated toward the null after additional adjustment for history of injury, medication use, and consultation (*P* quadratic = 0.17).

**Conclusions:**

This cross-sectional study showed that there were no significant linear or quadratic relationships of self-reported PA with CLBP and CKP. Future longitudinal study with objective measurements is needed.

## Abstract (ja)

**【背景】:**

身体活動の不足あるいは過剰な実施は慢性的な運動器の痛みと関連している可能性がある。本研究の目的は、身体活動量と慢性腰痛および慢性膝痛の関係を明らかにすることである。

**【方法】:**

地域在住の40から79歳住民を対象として、2009年に島根県で横断的調査を実施した。本研究では、その回答者4559人のデータについて解析を行った。自記式質問紙によって、社会人口統計学的変数および健康に関する項目を調査した。身体活動量は国際身体活動質問票（International Physical Activity Questionnaire）によって、慢性腰痛および膝痛は 改訂版Knee Pain Screening Toolを用いて評価した。身体活動量と慢性腰痛・膝痛有病率の関係は他の要因を調整したポアソン回帰分析によって検討した。

**【結果】:**

慢性腰痛と慢性膝痛の有病率はそれぞれ14.1%と10.7%であり、過去の受傷、医薬品の使用、医療機関の受診と有意に関連していた。共変量で調整する前・後ともに、身体活動量と慢性腰痛・膝痛との間に有意な関連は見られなかった（P>0.05）。性、年齢、教育年数、健康度自己評価、抑うつ症状、喫煙、慢性疾患既往歴、体格指数(Body-Mass Index)で調整した慢性膝痛の有病率比は、中程度の身体活動量（8.25-23.0 MET-時/週）の群と比較した場合、身体活動量が最も低い群で1.12（95%信頼区間、0.84-1.50）であり、 最も高い群で1.26 (0.93-1.70)であった（2次トレンドP値＝0.08）。これらの有病率比は、過去の受傷、医薬品の使用および医療機関の受診を調整変数に加えると、さらに１へと近づき縮小した（2次トレンドP値＝0.17）。

**【結論】:**

本研究では、自己申告による身体活動量と慢性腰痛・膝痛の間に、1次（直線的）および2次（曲線的）いずれの形でも有意な関係が見られなかった。客観的な評価指標を用いた縦断的研究による今後の検証が望まれる。

## INTRODUCTION

Musculoskeletal disorders are a major burden on individuals, health systems, and society, contributing meaningfully to indirect costs^1^ and disability worldwide.^2^ Further, chronic musculoskeletal pain (CMP), a major symptom of musculoskeletal disorders,^3^^–^^6^ worsens quality of life and physical functioning later in life.^7^^,^^8^ In the United States, 28.8% of men and 26.6% of women reported feeling some pain.^9^ The lifetime risk of low back pain in Japan is estimated to be 83%.^10^ However, despite its importance to public health, evidence linking lifestyle to CMP remains to be established.

Physical activity (PA), including exercise therapy, is recommended as a non-pharmacological intervention for CMP.^11^^,^^12^ Pharmacological treatments, including nonsteroidal anti-inflammatory drugs, are also commonly prescribed. Considering the expense of prescriptions and side effects of such treatments,^13^ increasing PA should receive greater priority both as a therapeutic agent and as preventative action against CMP. However, the relationship between PA levels and CMP has not been established yet.

Recently, both too little PA and too much PA were found to be hazardous to spinal health,^14^^,^^15^ indicating a U-shaped relationship between PA and chronic low back pain (CLBP). However, few studies have examined the dose-response relationship between PA and CMP.^15^^–^^18^

When examining the relationship between PA and CMP, weight status and musculoskeletal injury need to be accounted for, since adiposity is an established risk factor for knee osteoarthritis and CMP.^19^^–^^22^ Among overweight individuals, excessive PA may cause high physical load on the knee joint, leading to chronic knee pain (CKP).^23^ This mechanism suggests that excess PA may cause CMP, especially among overweight adults. Injury is also an established risk factor for CMP.^23^^,^^24^ Excess PA increases the probability of experiencing injury,^25^^,^^26^ and musculoskeletal injury may reduce PA levels,^27^^,^^28^ potentially leading to weight gain.^29^ For these reasons, it is important to consider both weight status and injury history when investigating the association of PA with CMP. To our knowledge, a history of injury has been accounted for only in studies examining the risk of knee osteoarthritis,^23^^,^^30^^,^^31^ while studies of the relationship between PA and CMP in the general population typically have not taken injury history into account. In addition, adults who have history of injury are likely to take medications and consult physicians, and these pain management factors may also affect pain itself as well as daily habits (such as PA). Thus, consideration of these factors is also important.

To fill the gap in knowledge on the potential role of PA in the development of CMP, we examined cross-sectional associations of PA with CLBP and CKP among adults in a community-based survey in Japan, taking into account potential confounding by body weight, history of joint injuries, and pain management factors. We also examined *post hoc* how these factors could influence associations between common demographic variables with CLBP and CKP.

## METHODS

### Data collection

We cross-sectionally evaluated observations from an ongoing community-based intervention study for community-level improvement in levels of PA.^32^ In October 2009, invitation letters, consent forms, and questionnaires were mailed to 6000 residents randomly selected from the city registry in Unnan City (population 43 520, area 553.4 km^2^), a rural mountainous region in Shimane, Japan. Men and women aged 40 to 79 years were invited to participate; excluded were those in assisted living facilities, those who required long-term care, and those who could not complete the questionnaires by themselves. We took a pragmatic approach to increase our survey response rate, including the use of personalised and relatively short questionnaires^33^ and sending postcard reminders to non-responders.

A total of 4559 adults (76.0%) responded to the initial survey of the trial and were considered eligible for the present study. Written informed consent was obtained from each participant. This study was approved by the research ethics committee of the Physical Education and Medicine Research Center UNNAN (H21-10-13-1).

### Measures

Sex and age were derived from the city registry, and other sociodemographic variables were obtained from self-administered questionnaires. We inquired about weight and height (used for calculating body mass index [BMI] in kg/m^2^), years of education, self-rated health (very good, good, poor, or very poor),^34^ depressive symptom (yes or no),^35^ smoking (never, past, or current), and chronic disease history (hypertension, hyperlipidaemia, diabetes, hyperuricaemia, stroke, heart disease, kidney and urologic diseases, liver disease, gastrointestinal disease, endocrine disease, or cancer). These covariates were selected because they previously have been reported to be associated with PA, musculoskeletal morbidity, or both.^23^^,^^36^

### Musculoskeletal pain

CLBP and CKP were assessed using a questionnaire (available as web-only supplemental material eQuestionnaire 1) that has questions similar to those in the Knee Pain Screening Tool (KNEST), except for questions about use of health services (which were not examined in this study).^37^^,^^38^ The KNEST was previously developed to screen and identify individuals who have knee pain in a general population. CLBP and CKP were defined as current pain (ie, episodes of pain at the time of the questionnaire) that had lasted longer than 3 months in the past year.^39^ We assessed the test-retest reliability of CLBP and CKP in study subjects by mailing the questionnaire twice to 500 randomly-selected adults aged 40–84 years, separated by an interval of 10 days. These were individuals living in Unnan who were not invited to participate in the main trial/survey. Evaluating the 206 respondents (response rate 41.2%; mean and standard deviation [SD] of age 63.4 and 11.9 years; 51.4% women) to both questionnaires, we observed moderate reliability (Cohen’s kappa 0.49 for CLBP and 0.72 for CKP).

We also obtained information on a visual analogue scale (VAS) for intensity of pain. We defined “severe chronic pain” as chronic pain with a VAS pain score ≥75 on a 100-point scale.^40^ However, the prevalence of severe chronic pain was low (low back: *n* = 96, 2.4%; knee: *n* = 83, 2.0%) in this general population. Thus, we were unable to analyze this outcome in detail in the current study. We also asked about a history of low back injury and knee injury, medication use, and consultation with physicians for low back or knee pain. These factors were included in analyses as dichotomous variables (yes or no for each item).

### Physical activity

We used the Japanese short version of the International Physical Activity Questionnaire (IPAQ),^41^ for which external reliability and validity have been reported elsewhere.^42^^,^^43^ The IPAQ asks separate questions about time spent on walking, moderate physical activity (MPA), and vigorous physical activity (VPA) in a typical week.

We estimated total weekly PA by multiplying the reported duration (hours) per week of walking, MPA, and VPA by their Metabolic Equivalent of Tasks (METs; walking = 3.3 METs; MPA = 4.0 METs; and VPA = 8.0 METs) to obtain estimated energy expenditure in MET-hours per week.^41^ Using these values, total moderate-to-vigorous physical activity (MVPA) was defined as 7 days × (3.3 METs × walking hours/day + 4.0 METs × MPA hours/day + 8.0 METs × VPA hours/day). The internal reliability over 10 days of the IPAQ was tested within our study, and found to be acceptable (Spearman correlation *r* = 0.64 among adults aged 40–84 years in the forementioned reliability study). In a validation study conducted among a sample of 95 subjects (40 men and 55 women) aged 62 to 85 (mean [SD], 74.9 [4.5]) years living in Unnan, we compared energy expenditure derived from the IPAQ with that objectively measured by a uniaxial accelerometer (Lifecorder, Suzuken Co., Ltd., Nagoya, Japan^44^^,^^45^). The validity (*r* = 0.33) was comparable to that observed in other studies.^42^^,^^43^

### Statistical analyses

We compared the prevalence of CLBP and CKP in adults with different PA levels, estimating prevalence ratios (PR) by multivariable-adjusted Poisson regression.^46^ Poisson regression was used because the prevalence of CLBP and CKP was relatively high (>10% each). We examined CLBP and CKP separately as well as simultaneously using generalized estimating equations because these outcomes were correlated (kappa = 0.20).^47^

We evaluated total MVPA levels both continuously and categorically. To define categories, we chose an MVPA cutpoint of 8.25, corresponding to the WHO recommendation of 2.5 hours/week of MVPA (brisk walking in this case).^48^ For those with ≥8.25 MET-hours/week, we used tertiles within this sufficiently active group to determine further cutpoints (23.1, 75.4). Thus, the participants were divided into five categories: 0, 0.01–8.24, 8.25–23.00, 23.01–75.39, and ≥75.40 MET-hours/week. The adjusted PR and 95% confidence intervals (CIs) were then estimated using the middle category (8.25–23.0 MET-hours/week) as the reference category to assess potential non-linear relationships between MVPA and CMP.

When we evaluated MVPA as a continuous variable, we truncated the variable at the 95th percentile value (180 MET-hours/week) and log-transformed the variable to minimize effects of outliers and right-skewed distribution; analyses without truncation and log-transformation produced similar results, although whether the homoscedasticity assumption was met was uncertain (data not shown). In the regression analyses, we separately tested linear and quadratic relationships between MVPA and CMP.

We adjusted for the following potential confounders: sex, age, years of education, self-rated health, depressive symptoms, smoking habit, and chronic disease history (Model 1). In a separate model, we further adjusted for BMI (Model 2), past history of joint injuries (Model 3), and medication use and consultation with physicians (Model 4). Prevalence ratios by each covariate were additionally evaluated. We also assessed whether excess PA was associated with CKP, especially among adults with greater weight, by testing for an interaction between MVPA and BMI for CKP prevalence, and by examining joint categories of BMI (<20, 20–24.9, and ≥25 kg/m^2^) and MVPA (<8.25, 8.25–39.59, and ≥39.6 MET-hours/week). For these analyses, we used the median value of MVPA in adults with sufficient PA (39.5 MET-hours/week) for the cutpoint. We further assessed interactions between MVPA and history of injuries (low back or knee) for the combined outcome of either CLBP or CKP. While a prior review recommended exclusion of adults previously experiencing joint injuries in such analyses,^23^ our sample size would have been substantially reduced by excluding adults with a history of injury (33% of total). In a sensitivity analyses, we examined only adults without such a history and findings were little changed. Thus, in the present analyses, we included them, treating history of injury as a potential confounder and an effect-modifier.

We examined the associations of the different PA intensities with CLBP and CKP. In these analyses, VPA, MPA, and walking (in minutes per week) were entered into the same model simultaneously. Categorical and continuous analyses were performed separately for each PA intensity.

Missing information was imputed to minimize bias due to missing information and repeated four times, under the assumption that values were missing at random.^49^^,^^50^ Each imputation was based on regression models including variables used in the main regression analyses. The five imputed datasets were analysed independently and combined for inference, accounting for variability of imputation.^49^^,^^50^ We also repeated our analyses evaluating adults with complete information only, including 3329 participants. Analyses (two-sided α < 0.05) were carried out using SAS version 9.3 (Cary, NC, USA).

## RESULTS

Of the 4559 participants, 46.3% were men, and participants had a mean (SD) age of 60.9 (10.6) years (Table 1). The median (interquartile range) level of MVPA was 10.6 (0–46.2) MET-hours/week. A total of 55% engaged in the recommended level of MVPA (≥8.25 MET-hours/week), whereas 25.6% did not engage in any MVPA. Adults with greater MVPA were more likely to be men, older, smokers, less educated, less depressed, and more likely to have prevalent chronic diseases and history of low back or knee injury (all *P* < 0.05); however, MVPA was not associated with BMI (*P* = 0.7) (data not shown).

**Table 1.** Characteristics of adults in a community-based survey in Shimane, Japan, 2009 (*n* = 4559)

	Total	Participants who had CLBP	Participants who had CKP
Number of participants	4559	605	471
Physical activity^a^			
MVPA, MET-hours/week	10.6 (0–46.2)	11.6 (0–49.5)	11.6 (0–56.3)
Vigorous physical activity, min/week	0 (0–0)	0 (0–0)	0 (0–10)
Moderate physical activity, min/week	0 (0–40)	0 (0–40)	0 (0–60)
Walking, min/week	120 (0–420)	120 (0–420)	123 (0–510)

Men, %	46.3	49.9	39.5
Age, years	60.9 (10.6)	62.8 (10.6)	65.9 (10.0)
40s, %	17.6	13.2	7.0
50s, %	26.8	24.3	20.4
60s, %	29.8	29.6	28.5
70s, %	25.8	32.9	44.2
Self-rated health			
Excellent or good, %	81.8	61.6	68.9
Education status, years	11.4 (2.4)	11.2 (2.4)	10.8 (2.3)
Chronic disease history, %^b^	62.0	68.4	64.8
Depressive symptom, %	47.6	52.4	72.8
Smoking			
Past smoker, %	8.8	11.4	9.2
Current smoker, %	16.9	18.9	9.6
Body mass index, kg/m^2^	22.5 (3.1)	22.7 (3.2)	23.6 (3.1)
Past low back injury, %	23.2	45.1	29.1
Past knee injury, %	16.0	24.1	42.5
Medication use for low back pain, %	18.5	50.2	35.5
Medication use for knee pain, %	11.8	20.6	51.0
Consultation with physicians for low back pain, %	16.3	43.7	26.9
Consultation with physicians for knee pain, %	11.6	17.7	53.0

CLBP, chronic low back pain; CKP, chronic knee pain; MET, metabolic equivalent; MVPA, moderate-to-vigorous physical activity.

Means (standard deviations) for continuous variables and proportions for categorical variables are presented unless stated otherwise.

^a^Median (interquartile range).

^b^Reporting history of any of the following diseases: hypertension, hyperlipidemia, diabetes, hyperuricemia, cerebrovascular disease, heart disease, kidney and urologic diseases, liver disease, gastrointestinal disease, endocrine disease, cancer.

**Figure 1.**
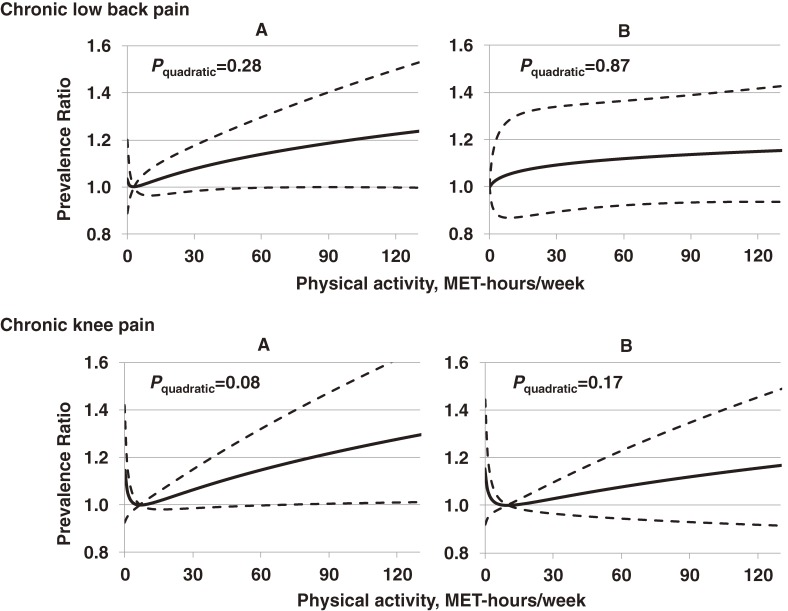
Associations between moderate-to-vigorous physical activity and the prevalence of chronic low back pain and chronic knee pain among Japanese adults (*n* = 4559). Solid lines represent prevalence ratios (PRs), and dashed lines indicate 95% confidence intervals estimated by Poisson regression, estimated by a quadratic function of physical activity levels (metabolic equivalent of task [MET]-hours/week). Panels on the left (A) display PR adjusted for sex, age, education years, self-rated health, depressive symptoms, smoking habit, chronic disease history, and body mass index; while on the right (B), PRs are further adjusted for history of joint injuries, medication use, and consultation with physicians for pain management. The reference value for each was fixed to the values giving the lowest prevalence of each outcome. *P* for each quadratic function is displayed.

CLBP was present in 14.1% of adults (*n* = 605), CKP was present in 10.7%, and both pain conditions were present in 3.7%. Fair or poor self-rated health, history of injury, medication use, and consultation with physicians were significantly associated with CLBP (Table 2). The relationship between MVPA and CLBP was not significant (*P* > 0.10 for both linear and quadratic associations). Although CKP was more prevalent in adults with the lowest (0 MET-hours/week) and the highest (≥75.4 MET-hours/week) PA (10.8% and 12.2%, respectively) than in those with average PA (9.7% in those with 8.25–23.0 MET-hours/week), PRs adjusted for potential confounders including BMI (Model 2) were not significantly different from 1.00 (lowest MVPA: PR 1.12, 95% CI 0.84–1.50; highest PA: PR 1.26, 95% CI 0.93–1.70) (Table 3). The non-significant quadratic association between PA and CKP (*P* = 0.08) in Model 2, further attenuated (to *P* = 0.17) in Model 4 after additional adjustment for history of injury and pain management (ie, medication use and consultation) (Figure 1). The pattern of results were similar to the above results with CLBP and CKP evaluated separately when we evaluated CLBP and CKP together as a combined outcome (*P* quadratic trend > 0.3; data not shown).

**Table 2.** Cross-sectional associations of energy expended on moderate to vigorous physical activity with chronic low back pain among Japanese adults (*n* = 4559)

	Adults with CLBP, %	PR (95% CI)^a^			

		Model 1^a^	Model 2^b^	Model 3^c^	Model 4^d^
PA levels, MET-hours/week					
0	14.9	0.94 (0.72–1.23)	0.93 (0.71–1.22)	0.95 (0.73–1.24)	0.93 (0.72–1.21)
0.1–8.24	12.8	0.86 (0.66–1.13)	0.86 (0.65–1.13)	0.89 (0.68–1.18)	0.86 (0.66–1.13)
8.25–23.0	15.0	1.0 (reference)	1.0 (reference)	1.0 (reference)	1.0 (reference)
23.1–75.3	13.7	0.94 (0.68–1.31)	0.94 (0.67–1.30)	0.95 (0.69–1.32)	0.98 (0.72–1.33)
≥75.4	16.1	1.09 (0.84–1.41)	1.10 (0.851.42)	1.04 (0.80–1.35)	1.02 (0.79–1.32)
*P* for linearity		0.14	0.13	0.30	0.20
*P* for quadratic		0.29	0.28	0.53	0.87
Sex, female	13.2	0.99 (0.82–1.19)	1.01 (0.84–1.21)	1.07 (0.89–1.28)	0.93 (0.78–1.12)
Age					
50s	12.5	1.16 (0.88–1.52)	1.16 (0.88–1.52)	1.18 (0.90–1.55)	1.13 (0.86–1.49)
60s	14.0	1.33 (1.00–1.78)	1.34 (1.01–1.79)	1.39 (1.05–1.86)	1.19 (0.89–1.60)
70s	19.0	1.62 (1.19–2.20)	1.64 (1.21–2.24)	1.63 (1.20–2.23)	1.26 (0.92–1.74)
Self-rated health, fair or poor	30.3	2.59 (2.18–3.08)	2.59 (2.18–3.08)	2.36 (1.98–2.81)	1.75 (1.46–2.09)
Education years, per year	—^e^	0.98 (0.94–1.03)	0.99 (0.95–1.03)	0.99 (0.95–1.03)	1.01 (0.96–1.05)
Chronic disease history	15.6	1.03 (0.86–1.22)	1.01 (0.85–1.21)	1.01 (0.85–1.21)	1.03 (0.86–1.23)
Depressive symptom	15.2	1.06 (0.90–1.26)	1.07 (0.90–1.27)	1.01 (0.86–1.20)	1.03 (0.86–1.23)
Smoking					
Past smoker	18.2	1.29 (0.98–1.70)	1.30 (0.99–1.71)	1.23 (0.93–1.62)	1.17 (0.88–1.54)
Current smoker	15.9	1.23 (0.98–1.55)	1.25 (0.99–1.57)	1.21 (0.96–1.52)	1.14 (0.91–1.44)
BMI, per 5 kg/m^2^	—^e^	—^f^	1.09 (0.97–1.23)	1.07 (0.95–1.22)	1.03 (0.91–1.17)
History of low back injury	27.6	—	—	2.38 (2.03–2.79)	1.60 (1.35–1.90)
Medication use for LBP	40.9	—	—	—	2.66 (2.17–3.27)
Consultation for LBP	39.8	—	—	—	1.88 (1.54–2.29)

BMI, body mass index; CI, confidence interval; CLBP, chronic low back pain; LBP, low back pain; MET, metabolic equivalent; PA, physical activity; PR, prevalence ratio.

^a^Model 1 adjusted for sex, age, education years, self-rated health, chronic disease history, depressive symptom, and smoking. Reference categories were male, 40s of age, excellent or good self-rated health, no chronic disease history, no depressive symptom, and never smoker. Linear and quadratic relationships were tested separately.

^b^Model 2 adjusted for variables in the Model 1 and BMI.

^c^Model 3 adjusted for variables in the Model 2 and history of joint injuries (two indicator variables for injury of the knee and of the low back; yes, no for each).

^d^Model 4 adjusted for variables in the Model 3 and pain management (medication use and consultation with physicians).

^e^Prevalence is not shown for continuous variable.

^f^Not included in the models.

**Table 3.** Cross-sectional associations of energy expended on moderate to vigorous physical activity with chronic knee pain among Japanese adults (*n* = 4559)

	Adults with CKP, %	PR (95% CI)^a^			

		Model 1^a^	Model 2^b^	Model 3^c^	Model 4^d^
PA levels, MET-hours/week					
0	10.8	1.15 (0.86–1.54)	1.12 (0.84–1.50)	1.14 (0.85–1.53)	1.14 (0.85–1.53)
0.1–8.24	9.9	1.02 (0.74–1.41)	0.99 (0.72–1.37)	0.98 (0.70–1.39)	0.98 (0.71–1.34)
8.25–23.0	9.7	1.0 (reference)	1.0 (reference)	1.0 (reference)	1.0 (reference)
23.1–75.3	10.3	1.09 (0.78–1.50)	1.06 (0.77–1.47)	1.03 (0.73–1.43)	0.97 (0.70–1.34)
≥75.4	12.2	1.26 (0.93–1.71)	1.26 (0.93–1.70)	1.19 (0.88–1.60)	1.15 (0.85–1.56)
*P* for linearity		0.53	0.43	0.79	1.00
*P* for quadratic		0.07	0.08	0.09	0.17
Sex, female	12.1	1.22 (0.98–1.52)	1.31 (1.05–1.64)	1.25 (1.00–1.56)	0.98 (0.79–1.22)
Age, years					
50s	8.0	1.85 (1.24–2.76)	1.88 (1.26–2.80)	1.84 (1.23–2.74)	1.51 (1.01–2.26)
60s	10.3	2.23 (1.49–3.32)	2.30 (1.54–3.44)	2.24 (1.50–3.34)	1.75 (1.16–2.62)
70s	19.1	3.77 (2.51–5.68)	4.14 (2.75–6.22)	3.56 (2.37–5.37)	2.06 (1.36–3.13)
Self-rated health, fair or poor	18.7	1.67 (1.36–2.06)	1.65 (1.34–2.03)	1.51 (1.22–1.86)	1.21 (0.98–1.49)
Education years	—^e^	0.96 (0.92–1.01)	0.97 (0.93–1.01)	0.97 (0.93–1.01)	1.01 (0.96–1.05)
Chronic disease history	12.7	1.18 (0.96–1.47)	1.07 (0.86–1.33)	1.06 (0.86–1.32)	0.98 (0.79–1.22)
Depressive symptom	11.2	1.19 (0.98–1.44)	1.24 (1.02–1.51)	1.20 (0.99–1.46)	1.17 (0.97–1.41)
Smoking					
Past smoker	11.1	1.11 (0.77–1.60)	1.15 (0.80–1.66)	1.17 (0.82–1.69)	1.17 (0.82–1.67)
Current smoker	6.0	0.73 (0.52–1.02)	0.78 (0.55–1.09)	0.80 (0.57–1.12)	0.87 (0.62–1.23)
BMI per 5 kg/m^2^	—^e^	—^f^	1.68 (1.47–1.91)	1.57 (1.37–1.80)	1.28 (1.11–1.48)
History of knee injury	29.0	—	—	3.23 (2.65–3.94)	1.67 (1.35–2.07)
Medication use for KP	49.4	—	—	—	2.99 (2.29–3.89)
Consultation for KP	51.5	—	—	—	3.11 (2.44–3.96)

BMI, body mass index; CI, confidence interval; CKP, chronic knee pain; KP, knee pain; MET, metabolic equivalent; PA, physical activity; PR, prevalence ratio.

^a^Model 1 adjusted for sex, age, education years, self-rated health, chronic disease history, depressive symptom, and smoking. Reference categories were male, 40s of age, excellent or good self-rated health, no chronic disease history, no depressive symptom, and never smoker. Linear and quadratic relationships were tested separately.

^b^Model 2 adjusted for variables in the Model 1 and BMI.

^c^Model 3 adjusted for variables in the Model 2 and history of joint injuries (two indicator variables for injury of the knee and of the low back; yes, no for each).

^d^Model 4 adjusted for variables in the Model 3 and pain management (medication use and consultation with physicians).

^e^Prevalence is not shown for continuous variable.

^f^Not included in the models.

Associations of age and history of injury with CLBP and CKP were found, but these associations attenuated when adjusted for medical treatment and consultation. A significant positive association of BMI with CKP, but not CLBP, persisted; per 5 kg/m^2^, PRs were 1.03 (95% CI 0.91–1.17) for CLBP and 1.28 (95% CI 1.11–1.48) for CKP, based on Model 4 (Tables 2 and 3). History of injury was also associated with each CMP outcome: PR 1.60 (95% CI 1.35–1.90) for CLBP and PR 1.67 (95% CI 1.35–2.07) for CKP (Tables 2 and 3).

**Figure 2.**
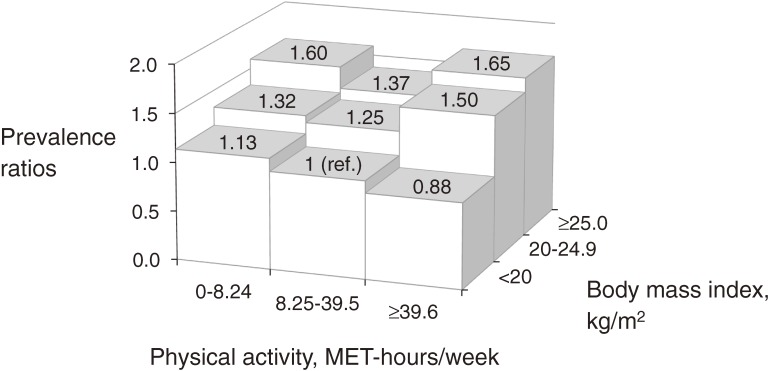
Associations of moderate-to-vigorous physical activity (metabolic equivalent of task [MET]-hours/week) and weight status with chronic knee pain among Japanese adults (*n* = 4559). Prevalence ratios were estimated with adjustment for sex, age, education years, self-rated health, depressive symptoms, smoking habit, chronic disease history, past joint injuries, medication use, and consultation with physicians for pain management. After adjustment, no significant prevalence ratios were observed (all *P* > 0.05). Interactions between body-mass index and physical activity levels in models, considering linear as well as non-linear associations, were also not significant (all *P* > 0.1).

The interaction between BMI and MVPA levels for CKP was not significant (*P* > 0.9 for linear and quadratic trends). When BMI and total MVPA levels were examined jointly, a non-significant U-shaped relationship between MVPA and CKP was observed in the high-BMI category (Model 4, Figure 2). The interaction between MVPA and joint injuries was also not significant (*P* = 0.88).

When we evaluated PA of different intensities, VPA, MPA, and walking were neither linearly nor non-linearly significantly associated with CLBP and CKP evaluated separately (all *P* > 0.05; data not shown) or with CLBP and CKP evaluated simultaneously as a combined outcome (Table 4).

**Table 4.** Cross-sectional associations between physical activity of different intensity and either chronic low back pain or chronic knee pain among Japanese adults (*n* = 4559)

Physical activity type	*n*	PR (95% CI)^a^			

		Model 1^b^	Model 2^c^	Model 3^d^	Model 4^e^
Vigorous PA, min/week					
0	3200	1.13 (0.80–1.59)	1.15 (0.81–1.63)	1.15 (0.83–1.59)	1.15 (0.91–1.45)
>0–40.6	453	1.0 (reference)	1.0 (reference)	1.0 (reference)	1.0 (reference)
40.9–180	458	1.27 (0.84–1.90)	1.26 (0.83–1.90)	1.18 (0.80–1.75)	1.19 (0.91–1.57)
>180	448	1.05 (0.67–1.66)	1.06 (0.67–1.67)	0.98 (0.64–1.49)	0.93 (0.67–1.29)
*P*_linearity_^f^		0.94	0.89	0.43	0.21
*P*_non-linearity_^f^		0.93	0.83	0.97	0.45
Moderate PA, min/week					
0	2990	1.05 (0.80–1.38)	1.02 (0.78–1.33)	1.04 (0.82–1.32)	1.16 (0.88–1.53)
>0–58.8	504	1.0 (reference)	1.0 (reference)	1.0 (reference)	1.0 (reference)
60.0–240	558	1.20 (0.87–1.65)	1.20 (0.88–1.63)	1.16 (0.87–1.55)	1.28 (0.96–1.69)
>240	507	1.13 (0.82–1.54)	1.14 (0.84–1.55)	1.13 (0.85–1.51)	1.29 (0.91–1.82)
*P*_linearity_^f^		0.18	0.07	0.17	0.22
*P*_non-linearity_^f^		0.56	0.61	0.52	0.28
Walking, min/week					
0	1271	1.10 (0.92–1.32)	1.11 (0.93–1.34)	1.09 (0.90–1.31)	1.08 (0.91–1.29)
>0–119	1055	1.0 (reference)	1.0 (reference)	1.0 (reference)	1.0 (reference)
120–404	1053	1.03 (0.85–1.25)	1.04 (0.86–1.26)	1.04 (0.86–1.26)	1.06 (0.88–1.26)
>404	1180	1.11 (0.91–1.36)	1.12 (0.91–1.38)	1.11 (0.90–1.36)	1.13 (0.93–1.36)
*P*_linearity_^f^		0.99	0.98	0.84	0.57
*P*_non-linearity_^f^		0.08	0.06	0.13	0.18

CI, confidence interval; PA, physical activity; PR, prevalence ratio.

^a^Prevalence ratios (PR) and 95% confidence intervals were estimated by Poisson regression. We examined chronic low back pain (CLBP), chronic knee pain (CKP), or both as the outcome of interest simultaneously by generalized estimating equation that accounted for the correlations between CLBP and CKP (kappa = 0.20). The models also included all PA measures simultaneously. Correlations among these PA categories were moderate (Spearman *r* = 0.48 between vigorous and moderate PA; 0.31 between vigorous PA and walking; 0.28 between moderate PA and walking). For each type of physical activity, we categorized adults into four groups by treating adults with 0 min/week as a single category and by splitting the others into tertiles.

^b^Model 1 adjusted for sex, age, education years, self-rated health, chronic disease history, depressive symptom, and smoking.

^c^Model 2 adjusted for variables in the Model 1 and body mass index.

^d^Model 3 adjusted for variables in the Model 2 and history of joint injuries (two indicator variables for injury of the knee and of the low back; yes or no for each).

^e^Model 4 adjusted for variables in the Model 3 and pain management (medication use and consultation with physicians).

^f^Log-linear and quadratic relationships were tested separately, using log-transformed variables.

All of these results from multiple imputed analyses were similar to those from complete-case analyses, with the exception of the complete-case analyses having less precision and wider confidence intervals; the variability of 5-time imputation was <10% of total variance (data not shown), while the variability due to multiple imputation was incorporated into estimations of precision and significant testing in all presented analyses.

## DISCUSSION

This study examined the associations of PA with CLBP and CKP among middle-aged and older Japanese. We found that there were no significant cross-sectional relationships of PA with CLBP and CKP. While neither a U-shaped association nor interactions by body mass and prior injury were statistically significant, our analysis indicate the importance of accounting for body mass, history of injury, medication use, and consultation with physicians in research on PA and CMP.

Few previous studies have examined a potential non-linear relationship between PA and CMP, especially for CKP. Some studies suggested U-shaped relationships between PA and CLBP.^15^^,^^17^^,^^18^ An occupational cohort study showed that the lowest and highest tertiles of minutes of MVPA yielded statistically significantly higher risks of low back pain than the middle tertile.^18^ However, our cross-sectional investigation did not detect any significant linear or quadratic associations of PA and CLBP or CKP.

Both positive and negative effects of excess PA on knee joint are conceivable. A systematic review concluded that there was strong evidence for an inverse relationship between PA and cartilage defects of the knee joint.^51^ However, the authors also concluded that there was a positive relationship between tibiofemoral osteophytes and PA. The results of previous studies on PA and joint health have been inconsistent, and many of the prior studies did not assess non-linear relationships or were too underpowered to do so.^51^ Therefore, future longitudinal investigations examining a potential non-linear relationship between PA and CMP are of value.

Our results also showed the importance of taking into account BMI, past injuries, and factors related to pain management, which were all significantly associated with CMP. Higher BMI level in this study was significantly associated with higher prevalence of CKP but not CLBP, in line with the postulation that a greater body mass causes physical burden on the knee joint.^23^ Our failure to show an interaction of PA and BMI on CKP may reflect the limited statistical power of the present study and also the limited range of BMI in our population, which predominantly comprised normal-weight adults with BMI < 25 kg/m^2^ (80%). Only a few prior studies took a history of injury into account.^18^^,^^23^ One third of the adults in our study reported a history of injury, and we observed a significant positive association of history of injury with CMP; it is possible that prior excess PA could have caused joint injury, which led to CMP. On the other hand, PA is recommended as a non-pharmacological intervention for CMP.^11^^,^^12^ Thus, adults who had history of injury, and possibly CMP, might engage in more PA for treatment and rehabilitation.

Our results showed that there were strong associations of CMP with medication use and consultation with physicians and that adjusting for these factors attenuated the quadratic association between PA and CKP. As seeking medications and undergoing outpatient treatment is directly associated with not only pain but also PA, these results are plausible. Our findings thus emphasize that future research on the relationship between PA and CMP should consider effects of BMI, injury, and pain management factors.

Globally, disability due to musculoskeletal disorders is estimated to have increased by 45% from 1990 to 2010, related to the aging of the population.^2^ It remains unknown what the most effective and affordable strategies are to reduce the global burden of musculoskeletal disorders.^52^ Although we detected little indication of benefits of PA for CMP, potential beneficial effects of PA on CMP still deserve discussion. Possible pathways linking greater PA to a reduced risk of CMP include but are not limited to reduction of mechanical stress through improving muscle strength, range of movement, and joint structure; improvement of blood flow to painful regions; relief of psychological stress, such as distraction and depression^7^^,^^53^^–^^55^; and elevation of tolerance to pain associated with increased serum concentrations of endocannabinoids that reduce pain sensation.^56^ Our community-based research in Japan, which has one of the most aged societies in the world, provides important insights into the studies on PA and musculoskeletal health.

Our study has several limitations. In our cross-sectional study, reverse causation and recall bias might have occurred. Individuals with CMP may reduce levels of recreational PA and PA intensities, leading to null findings for MVPA and CMP. Limitations are likely to be present in our assessment of injury, because this was ascertained retrospectively. We also had a limited sample size to tease out independent relations among PA levels, CMP, and potential confounders. Future research should adopt a longitudinal design, assessing PA prior to the development of injuries or pains. Considering potential biases due to self-reported PA, objective measures of PA, as well as anthropometrics, injuries, and pain, should be incorporated in future research.

In conclusion, this cross-sectional study showed that there were no significant linear or quadratic relationships of PA with CLBP and CKP. Our findings indicate the importance of evaluating PA, CMP, body mass, injuries, and pain management factors simultaneously.

## ONLINE ONLY MATERIALS

eQuestionnaire 1. Musculoskeletal pain questionnaire.

Abstract in Japanese.
