# Supplementary material for: Relationship Between Physical Activity and Chronic Musculoskeletal Pain Among Community-Dwelling Japanese Adults
Source: J Epidemiol. 2014 Nov 5;24(6):474–83. doi: 10.2188/jea.JE20140025 (PMC4213222; doi:10.2188/jea.JE20140025)
Supplement: eQuestionnaire 1. [file je-24-474-s001.pdf]

＜腰の痛みについてお聞きします。＞ [Low Back Pain Section]

1. これまでに、腰の痛みの経験がありますか？(Have you ever had pain in your low back?)

1．ない　2．ある
(1.　No　　2. Yes)

2. これまでに、医師の診察が必要になるような腰のケガをした経験がありますか？

(Have you ever injured your low back badly enough to see a doctor about it?)

1．ない　2．ある
(1.　No　　2. Yes)

3. <u>この1年間</u>、腰が痛むことがありましたか？

(Have you had pain in the last year in your low back?)

1．ない　2．あった
(1.　No　　2. Yes)

⇒あった方にお尋ねします。その腰の痛みはどのくらい続きましたか（続いていますか）？

(The following questions are specific to people who answer ‘yes’ to the above question. On how many days have you had this low back pain?)

1．7日未満　2．1～4週間　3．1ヶ月以上3ヶ月未満　4．3ヶ月以上
(1. Less than 7 days　2. 1-4 weeks　3. More than 1 month but less than 3 months　4. More than 3 months

⇒その腰の痛みは、<u>現在も</u>続いていますか？

(Do you still feel that low back pain?)

1．はい　2．いいえ
(1. Yes　　2. No)

4. この 1 年間、腰の痛みのために、受診することがありましたか？

(Have you consulted a doctor in the last year because of your low back pain?)

1．なし　2．1回　3．2～6回　4．7回以上
(1. No　2. Once　3. 2 to 6 times　4. 7 times or more)

5. 次の線は、**<u>この数日間（ここ 2、3 日）</u>の腰の痛みの程度**をおたずねするものです。 左の端を「痛みなし」、右の端を「最も激しい痛み」としたときに、この数日間のあなたの痛みの程度はどのあたりでしょうか？ **線の上でこのあたりと思われるところに×印をつけてください。**

(The following question will ask you about the degree of low back pain you experienced over the past few days. Responses may range from the far left side (“no pain at all”) to the far right (“the most severe pain you’ve ever had”). Mark an X indicating where you think the level of pain fell.)

|―――――――――――――――――――――――――|

痛みなし (no pain at all)　　　　これまでに経験した最も激しい痛み (the most severe pain you’ve ever had)

6. 現在、ヒザの痛みのために医薬品（飲み薬、湿布等）を使用していますか？使用している場合、何種類か数も書いてください。　※サプリメントは含みません。

(Are you currently taking any medication (oral medicine, analgesic plaster, etc.) because of your low back pain? If yes, please indicate the number of medications you take. *Do NOT include dietary supplements.)

1. はい（______種類）　2. いいえ
(1. Yes (#_____medications)　2. No)

＜ヒザ（膝）の痛みについてお聞きします。＞ [Knee Pain Section]

7. これまでに、ヒザの痛みの経験がありますか？(Have you ever had pain in the knee?)

1. ない　2. 右ヒザだけ　3. 左ヒザだけ　4. 両ヒザ
(1. No　2. Right knee only　3. Left knee only　4. Both knees)

8. これまでに、医師の診察が必要になるようなヒザのケガをした経験がありますか？

(Have you ever injured your knee badly enough to see a doctor about it?)

1. ない　2. 右ヒザだけ　3. 左ヒザだけ　4. 両ヒザ
(1. No　2. Right knee only　3. Left knee only　4. Both knees)

9. この１年間、ヒザやヒザのまわりが痛むことがありましたか？

(Have you had pain in the last year in or around the knee?)

1. ない　2. 右ヒザだけ　3. 左ヒザだけ　4. 両ヒザ
(1. No　2. Right knee only　3. Left knee only　4. Both knees)

⇒あった方にお尋ねします。そのヒザの痛みはどのくらい続きましたか（続いていますか）？

(The following questions are specific to people who answer ‘yes’ to the above question. On how many days have you had this knee pain?)

1. 7日未満　2. 1～4週間　3. 1ヶ月以上3ヶ月未満　4. 3ヶ月以上
(1. Less than 7 days　2. 1-4 weeks　3. More than 1 month but less than 3 months　4. More than 3 months)

⇒そのヒザの痛みは、現在も続いていますか？

(Do you still feel that knee pain?)

1. はい　2. いいえ
(1. Yes　2. No)

10. この 1 年間、ヒザの痛みのために、受診することがありましたか？

(Have you consulted a doctor in the last year because of your knee pain?)

1. なし　2. 1回　3. 2～6回　4. 7回以上
(1. No　2. Once　3. 2 to 6 times　4. More than 7 times)

11. 次の線は、**この数日間（ここ 2、3 日）のヒザの痛みの程度**をおたずねするものです。 左の端を「痛みなし」、右の端を「最も激しい痛み」としたときに、この数日間のあなたの痛みの程度はどのあたりでしょうか？ **線の上でこのあたりと思われるところに×印をつけてください。**

(The following question will ask you about the degree of knee pain you experienced over the past few days. Responses may range from the far left side (“no pain at all”) to the far right (“the most severe pain you’ve ever had”). Mark an X indicating where you think the level of pain fell.)

痛みなし (no pain at all) —— これまでに経験した最も激しい痛み (the most severe pain you’ve ever had)

12. 現在、ヒザの痛みのために医薬品（飲み薬、湿布等）を使用していますか？使用している場合、何種類か数も書いてください。　※サプリメントは含みません。

(Are you currently taking any medication (oral medicine, analgesic plaster, etc.) because of your knee pain? If yes, please indicate the number of medications you take. *Do NOT include dietary supplements.)

1. はい（______種類）　2. いいえ
(1. Yes (#_____medications)　2. No)
