# Supplementary material for: Relationship Between Physical Activity and Chronic Musculoskeletal Pain Among Community-Dwelling Japanese Adults
Source: J Epidemiol. 2014 Nov 5;24(6):474–83. doi: 10.2188/jea.JE20140025 (PMC4213222; doi:10.2188/jea.JE20140025)
Supplement: Abstract in Japanese. [file je-24-474-s002.pdf]

地域在住の日本人成人における身体活動と慢性運動器疼痛の関係

鎌田真光[1-4], 北湯口純[3], I-Min Lee[4], 濱野強[5], 今村文昭[6], 井上茂[7], 宮地元彦[1], 塩飽邦憲[8]

1 独立行政法人国立健康･栄養研究所健康増進研究部

2 日本学術振興会

3 身体教育医学研究所うんなん

4 Division of Preventive Medicine, Brigham and Women’s Hospital, Harvard Medical School

5 島根大学研究機構戦略的研究推進センター

6 MRC Epidemiology Unit, Institute of Metabolic Science, University of Cambridge School of Clinical Medicine

7 東京医科大学公衆衛生学講座

8 島根大学医学部環境予防医学

【背景】 身体活動の不足あるいは過剰な実施は慢性的な運動器の痛みと関連している可能性がある。本研究の目的は、身体活動量と慢性腰痛および慢性膝痛の関係を明らかにすることである。

【方法】 地域在住の 40 から 79 歳住民を対象として、2009 年に島根県で横断~~的~~調査を実施した。本研究では、その回答者 4559 人のデータについて解析を行った。自記式質問紙によって、社会人口統計学的変数および健康に関する項目を調査した。身体活動量は国際身体活動質問票（International Physical Activity Questionnaire）によって、慢性腰痛および膝痛は 改訂版 Knee Pain Screening Tool を用いて評価した。身体活動量と慢性腰痛・膝痛有病率の関係は他の要因を調整したポアソン回帰分析によって検討した。

【結果】 慢性腰痛と慢性膝痛の有病率はそれぞれ 14.1%と 10.7%であり、過去の受傷、医薬品の使用、医療機関の受診と有意に関連していた。共変量で調整する前・後ともに、身体活動量と慢性腰痛・膝痛との間に有意な関連は見られなかった（P＞0.05）。性、年齢、教育年数、健康度自己評価、抑うつ症状、喫煙、慢性疾患既往歴、体格指数(Body-Mass Index)で調整した慢性膝痛の有病率比は、中程度の身体活動量（8.25-23.0 MET-時/週）の群と比較した場合、身体活動量が最も低い群で 1.12（95%信頼区間、0.84-1.50）であり、 最も高い群で 1.26 (0.93-1.70)であった（2 次トレンド P 値＝0.08）。これらの有病率比は、過去の受傷、医薬品の使用および医療機関の受診を調整変数に加えると、さらに1へと近づき縮小した（2 次トレンド P 値＝0.17）。

【結論】 本研究では、自己申告による身体活動量と慢性腰痛・膝痛の間に、1 次（直線的）および2次（曲線的）いずれの形でも有意な関係が見られなかった。客観的な評価指標を用いた縦断的研究による今後の検証が望まれる。

キーワード： 運動、運動器疼痛、関節炎、疫学、公衆衛生
